# Pre-hospital care time intervals among victims of road traffic injuries in Iran. A cross-sectional study

**DOI:** 10.1186/1471-2458-10-406

**Published:** 2010-07-09

**Authors:** Maryam Bigdeli, Davoud Khorasani-Zavareh, Reza Mohammadi

**Affiliations:** 1 Urmia University of Medical Sciences, Urmia, Islamic Republic of Iran; 2 Division of Social Medicine, Department of Public Health Sciences, Karolinska Institutet, Norrbacka, SE-171 76 Stockholm, Sweden

## Abstract

**Background:**

Road traffic injuries (RTIs) are a major public health problem, requiring concerted efforts both for their prevention and a reduction of their consequences. Timely arrival of the Emergency Medical Service (EMS) at the crash scene followed by speedy victim transportation by trained personnel may reduce the RTIs' consequences. The first 60 minutes after injury occurrence - referred to as the "golden hour"- are vital for the saving of lives. The present study was designed to estimate the average of various time intervals occurring during the pre-hospital care process and to examine the differences between these time intervals as regards RTIs on urban and interurban roads.

**Method:**

A retrospective cross-sectional study was designed and various time intervals in relation to pre-hospital care of RTIs identified in the ambulance dispatch centre in Urmia, Iran from 20 March 2005 to 20 March 2007. All cases which resulted in ambulance dispatches were reviewed and those that had complete data on time intervals were analyzed.

**Results:**

In total, the cases of 2027 RTI victims were analysed. Of these, 61.5 % of the subjects were injured in city areas. The mean response time for city locations was 5.0 minutes, compared with 10.6 minutes for interurban road locations. The mean on-scene time on the interurban roads was longer than on city roads (9.2 vs. 6.1 minutes, p < 0.001). Mean transport times from the scene to the hospital were also significantly longer for interurban incidents (17.1 vs. 6.3 minutes, p < 0.001). The mean of total pre-hospital time was 37.2 (+/-17.2) minutes with a median of 32.0. Overall, 72.5% of the response interval time was less than eight minutes.

**Conclusion:**

The response, transport and total time intervals among EMS responding to RTI incidents were longer for interurban roads, compared to the city areas. More research should take place on needs-to and access-for EMS on city and interurban roads. The notification interval seems to be a hidden part of the post-crash events and indirectly affects the "golden hour" for victim management and it needs to be measured through the establishment of the surveillance systems.

## Background

Road traffic injuries (RTIs) are a major public health problem, requiring concerted efforts for prevention [1,2]. The best strategy for RTI control is crash prevention, however total prevention is obviously impossible and crashes can occur at any time [2-4]. However, it is often possible to minimize crash consequences by promptly providing effective pre-hospital services [4,5]. Each year, many of the 1.2 million lives lost globally could be saved and much of the ensuing disability suffered by the 50 million injured could be prevented if rapid and competent pre-hospital services were available at the crash scene [1,6].

In most low-and middle-income countries (LMICs), transport of road traffic victims, is usually provided by relatives, taxi drivers, truck drivers, police officers and other motorists; who are usually untrained [7,8]. Ambulances, if available, usually exist only in urban areas [6]. Significant numbers of neurological injuries appear to be a result of the extrication process or victim transportation without adequate immobilization [3,9,10], generally by untrained people [11]. Studies have shown that the inadequacy of public health infrastructure and poor access to health services are important reasons for the high burden of RTIs and/or their severity [3]. Many LMICs have insufficient pre-hospital emergency medical services including rapid services and effective management of RTI victims and their transportation [12] and therefore their improvement and system evaluation is crucial [13,14].

Pre-hospital care is unsatisfactory in many countries, especially in LMICs [7-9], where the majority of trauma deaths occur in the pre-hospital phase. Rapid arrival of the EMS at the crash scene and proper victim transportation by trained personnel may reduce injury severity and reduce the number of preventable deaths. It is important to note that many trauma experts consider that the first 60 minutes after injury occurrence - referred to as the "golden hour"- are the most effective for saving lives [15]. After this period, the risk of death or injury severity rises significantly [15]. This "golden hour" consists of various time intervals, e.g. notification interval, activation interval, response interval, on-scene interval, and transport interval (see Appendix 1).

Rapid responses are believed to be one of the most important criteria for the quality of care provided to trauma patients [15]. Measuring these various time intervals can be an important step towards the evaluation of the EMS function. However, to our knowledge there is a lack of information about the various pre-hospital time intervals of road traffic injuries and the differences between these for city and interurban roads area in Iran. The present study therefore was designed to estimate the average timings of various time intervals of RTI at the pre-hospital phase by EMS to trauma centres in the capital city of West Azarbaijan Province of Iran.

## Method

This is a retrospective cross-sectional study on time intervals of RTIs that were identified in the centre for ambulance dispatch sites from 20 March 2005 to 20 March 2007 in the Urmia city of Iran. The pre-hospital data of all RTIs were reviewed and the average of the different interval times was analyzed.

### Study area and study population

This study was undertaken in Urmia, the capital city of the West Azarbaijan Province of Iran, which is located in the centre of the province. West Azarbaijan province shares a common border with Iraq, Turkey and Russia Azerbaijan. For feasibility reasons Urmia was chosen for the study. The population was about 887 318 in 2006. The rate of fatal RTIs in this province was estimated at 34 per 100 000 in 2005 [16].

### Data collection instrument

**Figure 1:**
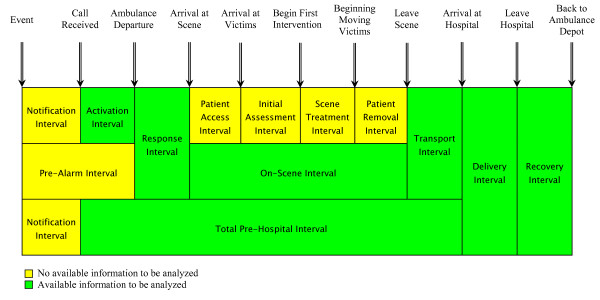
**Specific intervals and points in time for road traffic injury victims**. Adapted with some modification from references [15,25].

A standard questionnaire designed by the Ministry of Health and Medical Education in Iran, was used in this study. The questionnaire has demographic information on patients or victims including name, sex and age, disease or external cause of injury and information about time of services including time of emergency call, time of ambulance departure to scene, time of arrival at scene, time of patients' transportation, time of arrival at hospital, time of leaving the hospital and time of returning to ambulance depot, location of crash scene (in cities or on interurban roads), as well as distance covered by the ambulance from the departure to return to depot. In this study, time intervals including time of emergency call, time of ambulance departure to scene, time of arrival at scene, time for patients' transportation and time of arrival at the hospital were used. Figure 1 illustrates these different time intervals.

### Data source and case selection

Trained EMS ambulance personnel record information about patients/or victims of RTIs. Ambulance personnel are responsible for death and injury registration. For each patient there is a questionnaire to be filled out (see also data collection). After data collection by technicians, these data will be fed into a central computer located at the ambulance site dispatch centre by a trained technician. Initially, all calls to the ambulance centre were reviewed. In total, there were 22 182 registered calls to the EMS in Urmia that resulted in EMS activity. Among all calls during the study period, 2 210 were related to RTIs. The inclusion criteria were, if they qualified as RTI victims and resulted in a ground ambulance dispatch; and received service from them, e.g. they were transported from the scene by one of the region's EMS. Incident locations were defined as "city" and "interurban roads" if they occurred in the Urmia geographic area, according to the Statistical Centre of Iran definition. The cases lacking in complete information about time intervals were excluded (183 cases). In total, 2027 cases were analyzed in this study.

### Data treatment

Descriptive analysis on various pre-hospital time intervals including: activation interval, response interval, on-scene interval, transportation interval and the total pre-hospital intervals were investigated, using mean, median, mode, maximum, minimum and 95% confidence intervals. Moreover, bivariate analyses were conducted for time intervals (five categories) and crash location (two categories) using t-tests and Chi^2 ^test, to detect significant association and differences (P < 0.05) in distribution between categorical and continuous variables, respectively. Moreover, the distribution of time of injury occurrence (four categories for time of injury; seven categories for date of injury; and four categories for season of injury) was considered for city and interurban roads, using Chi^2 ^test. In order to test the difference between weekdays and seasons of injury occurrence in relation to crash location, the t-test used again. The SPSS version 13.00 (SPSS Inc, Chicago, IL, USA) was used for data analysis.

The study was approved by the Iranian National Ethics Committee at the Ministry of Health and Medical Education of Iran. Permission was also obtained from Urmia University of Medical Sciences.

## Results

From all ambulance dispatches by EMS, 61.5% of RTIs victims were injured within the city compared to 38.5% cases that occurred on interurban roads. Among them, 27.7% deaths occurred in the city and the rest on interurban roads. Overall, 1.8% of all subjects died after EMS arrival at crash scene or en route to hospitals.

### Time intervals of EMS activities

The mean values for the different time intervals of the activities are summarized in Table 1. The mean of the response interval and on-scene interval were approximately the same. The transport interval was slightly longer than response intervals. The variation of the total time interval was long with a minimum and maximum of 14-114 minutes, respectively.

**Table 1:** Mean, median, mode, minimum and maximum of the time intervals of road traffic injury in the Urmia from 20 March 2005 to 20 March 2007.

Time intervals	Mean (SD)	Median	Mode	Min	Max	95% CI
Activation interval	1.4 (0.59)	1	1	1	8	1.39-1.46
Response interval	7.1 (5.6)	6	2	2	45	6.87-7.49
On-scene interval	7.4 (5.2)	6	5	3	25	7.06-7.64
Transport interval	10.5 (9.1)	7	5	3	57	9.96-10.95
Total time intervals	37.2 (17.2)	32	26	14	114	36.25-38.18

Table 2 presents time intervals by measurements of the central tendency in the EMS, stratified by city and interurban roads. The mean response time on interurban roads was longer than within the city (10.6 minutes vs. 5.0 minutes, p < 0.001). Moreover, the on-scene interval was longer on interurban roads compare to the city (9.2 vs. 6.1 minutes, p < 0.001). The transport time interval for both city and interurban roads was slightly longer than the response time interval. The mean of total pre-hospital time was almost twice for interurban roads compared to the city (p < 0.001).

**Table 2:** Pre-hospital time intervals of road traffic injury stratified by crash location in the Urmia from 20 March 2005 to 20 March 2007.

	City		Interurban roads	
	
Time intervals (minute)*	Mean (SD)	Median (0.25%-0.75%)	Mean (SD)	Median (0.25%-0.75%)
Activation interval	1.4 (0.6)	1 (1-2)	1.4(0.6)	1 (1-2)
Response interval	5.0 (3.1)	4 (3-7)	10.6 (7.0)	9 (6-13)
On-scene interval	6.1(3.7)	5 (4-7)	9.2 (6.7)	7 (5-11.3)
Transport interval	6.3 (3.6)	5 (4-8)	17.1(10.9)	14 (9-23)
Total pre-hospital	29.2 (9.1)	28 (22-34)	49.9 (19.4)	45 (35-62)

*Despite of activation interval, all P-values were < 0.001

As Table 3 shows, there was no significant association between activation time for RTIs on city and interurban roads. However, there was significant association for response time in the city compared to on interurban roads (p < 0.001). Focusing on total pre-hospital time intervals, around eight out of ten victims in the city were transported in less than 30 minutes, while for victims on interurban roads; one out of four had been transported in less than 30 minutes.

**Table 3:** Proportion of the different time intervals of road traffic injury stratified by crash location in the Urmia from 20 March 2005 to 20 March 2007

	City	Interurban roads	Total
Time intervals (minute)	N = 1246	N = 781	N= 2027
***Activation interval***	**%**	**%**	**%**
**≤ 2**	98.1	99.2	98.5
**> 2**	1.9	0.8	1.5
Chi-square= 0.9; P = 0.29			
***Response interval***			
**< 8**	89.2	45.7	72.5
**8-15**	9.4	34.3	18.9
**> 15**	1.4	20.0	8.6
Chi-square= 301.3; P < 0.001			
***On-scene interval***			
**< 5**	51.2	37.2	45.8
**5 -10**	39.5	34.3	37.5
**> 10**	9.3	28.5	16.7
Chi-square= 82.9; P < 0.001			
***Transportation interval***			
**< 10**	88.8	34.0	67.7
**10-20**	10.7	37.5	21.0
**> 20**	0.5	28.5	11.3
Chi-square= 450; P < 0.001			
***Total interval***			
**< 30**	79.5	25.1	58.5
**30-45**	17.2	30.6	22.4
**> 45**	3.3	44.3	19.1
Chi-square= 454; P < 0.001			

Response times varied significantly between the city compared to interurban roads (P < 0.001). Close to 90% of response time within the city were less than 8.0 minutes, compared to 45.7% on interurban roads. There was significant association between transportation interval time and crash site (P < 0.001). In general, 99.5% of victims of RTIs in the city had arrived at hospital within 20 minutes of departure from the crash scene. Close to half of the cases on interurban roads reached the hospital more than 45 minutes after departure (P < 0.001).

As Table 4 shows, seven out of ten of the injuries occurred between 8.00 am and 8.00 pm. There was an association between time of injury occurrence and crash site (P < 0.001); of which close to half of the injuries on interurban roads occurred between 14:01-20:00, which at the same time this is different from the injury occurrence in the city area. There was an association between the victims' crash site and the days of the week (P = 0.02).

**Table 4:** Proportion of the time of RTIs occurrence at city and interurban roads in the Urmia from 20 March 2005 to 20 March 2007

	City	Interurban roads	Total
	
Time, date and season of injury	N = 1246	N = 781	N= 2027
***Time of injury***	**%**	**%**	**%**
08:01-14:00	33.0	29.8	31.7
14:01-20:00	35.3	48.2	40.3
20:01-02:00	26.1	16.7	22.5
02:01-08:00	5.6	5.3	5.5
	Chi-square= 25.1; P < 0.001		
***Date of injury***			
Sunday	13.9	14.2	14.0
Monday	13.6	10.1	12.2
Tuesday	14.8	14.8	14.8
Wednesday	13.3	14.0	13.6
Thursday	16.5	12.1	14.8
Friday	15.1	21.8	17.7
Saturday	12.8	13.0	12.9
	Chi-square= 14.8; P = 0.02		
***Season of injury***			
Spring	33.5	34.8	34.0
Summer	28.8	26.1	27.8
Autumn	20.9	24.1	22.1
Winter	16.8	15.0	16.1
	Chi-square= 3.0; P = 0.3		

The effect of the date and season of injury on the mean time intervals (response time, transport time and total time interval) of the ambulance dispatches was also explored in this analysis. As Table 5 shows, there was an association between the mean of the above time intervals during weekdays at crash location (P < 0.05). However, none of the time intervals varied significantly between city and interurban roads areas, when the time intervals were stratified according to season (P < 0.05).

**Table 5:** Mean of the time intervals on city and interurban roads stratified by date, season and crash occurrence in the Urmia from 20 March 2005 to 20 March 2007

	Mean(minute)*	
	
Date and season of injury	City	Interurban roads
**Weekday**		

***Response time***		
Sunday	5.1	8.9
Monday	5.0	10.8
Tuesday	4.7	11.3
Wednesday	4.5	11.1
Thursday	5.6	10.5
Friday	4.9	11.2
Saturday	4.8	9.9
***Transport time***		
Sunday	5.7	13.8
Monday	6.9	16.3
Tuesday	6.0	17.6
Wednesday	6.6	18.9
Thursday	6.5	15.6
Friday	5.3	23.7
Saturday	6.1	18.6
***Total pre-hospital time***		
Sunday	25.6	40.2
Monday	25.4	49.3
Tuesday	23.9	48.2
Wednesday	24.5	47.9
Thursday	26.5	44.1
Friday	23.8	52.1
Saturday	24.2	45.8

**Season**		
***Response time***		
Spring	5.0	9.6
Summer	4.7	10.5
Autumn	5.0	11.4
Winter	5.3	11.8
***Transport time***		
Spring	6.5	17.2
Summer	5.5	19.3
Autumn	5.9	17.8
Winter	7.0	20.1
***Total pre-hospital time***		
Spring	25.1	44.5
Summer	23.4	44.9
Autumn	25.2	48.1
Winter	26.5	56.2

*P values for all were < 0.05

## Discussion

This study estimated the various time intervals for ambulance services and the differences between them for city against interurban roads. The time intervals in this study can be important indicators for EMS performance evaluation in terms of resource planning and maybe also useful for assessing quality of patient care. Most of the time intervals were lower than similar studies in Iran. The time interval between the RTI occurrence and the onset of care at a designated trauma centre has been thought to be an important predictor of victim survival [17,18].

The average response interval in this study was lower than the findings from the capital city of Iran (7.1 vs. 14.9 minutes). The reasons for this shorter interval compared to Tehran, are mainly related to the infrastructure of the study area [16]. Urmia is a small city compared to Tehran, which is a very big city and usually suffers from traffic congestion that can result in delays in response time. Focusing on the location of the injury occurrence, the response interval for interurban roads was longer than in the city. Other studies have revealed that rapid responses are believed to have a major effect on the quality of care provided to trauma patients. If time to the trauma centre is a critical variable in the prediction of trauma outcome; then planners of emergency medical services on interurban road areas may be faced with the difficult task of providing services to victims in this geographic location in terms of both pre-hospital care and hospital-based care, that is in line with line with previous recommendation in other studies [19,20].

The on-scene interval in this study was shorter than findings in Tehran, 7.4 vs. 18.0 minutes [21]. This time interval mainly can be affected by the skill of EMS team members as well as the involvement of lay people at the crash scene. In recent years many activities have been carried out in the study area [22] aimed at enhancing EMS personnel skills, which has resulted in their better performance during victim rescue. The other reason for such a short interval could also be related to the involvement of members of the public, initially as first responder and then their sense of urgency regarding victim transportation before ambulance arrival. A study in Iran has indicated that, untrained laypeople feel that removing victims from the crash scene and taking them to hospital quickly is the best course of action for their survival [22]. This can result in over-rapid extrication of the casualties from the trapped vehicles before the arrival of the ambulance crew. It is also important to note that, since motor vehicle crashes on interurban road areas often involve high speeds resulting in more serious crashes and injuries and they may require longer extrication times, a result which is line with Grossman et al. [19].

The transport interval in this study was shorter than at other settings in Iran, 10.5 minutes compare to 18.5 in Tehran [23]. The reason for this again may mainly be related to the infrastructure of the city and the availability of different hospitals combined with low traffic congestion; factors which can provide better conditions for victim transportation. It is important to note that the response time interval on interurban roads was significantly longer than that within the city. However, compared to the transport interval, this variation was lower. This may imply, on the one hand, that the location of the ambulance dispatch sites allows ambulances to arrive at crash scenes more quickly and hence provide better ambulance access to victims, but, on the other hand, that it takes longer for them to transport victims to hospital. As a result of this comparison, information regarding the effect of time on outcomes may be helpful in decisions on the geographic location of transport and first response teams as well as the sites for ambulance dispatch.

In general, the total pre-hospital time interval in this study was 37.2 minutes. Focusing on this time interval, when it is stratified by crash occurrence in the city and on interurban roads, it was 29.2 and 45.0 minutes, respectively. Compared to a study in Tehran, our finding was low, 37.2 vs. 45.0 minutes. As explained earlier regarding the response time interval, the same reason could be considered for this difference. Moreover, in recent years, the EMS have dramatically improved in the country as a whole [24], including an increase in the number of ambulances and ambulance dispatch sites, improvements in the equipment, and educational plans for emergency team staff [22]. All these factors can affect the shorter response as well as total pre-hospital time intervals.

As Calland (2005) noted that " the term Golden Hour, i.e. the first 60 minutes after crash occurrence, was first introduced in 1961, but due to misinterpretation as to what period this actually referred to, a second concept, the "Platinum Ten Minutes" was proposed as the time taken to move a victim to the ambulance. To achieve this rapid removal, the ambulance and medical personnel must work in harmony with the police and fire service to secure the scene and remove the RTI victims safely without causing injury either to the casualty or other personnel on the scene".

Currently there is no available information about the notification interval in this study, however as Figure 1 shows, this time interval is a hidden part of the total interval and it is crucial to the eventual outcome of each dispatch. This interval may be affected by many factors other than improvement in the pre-hospital care. A study in Tehran revealed that the mean time between the crash occurrence and arrival at the hospital was 170 minutes, while the total time interval from notification to arrival hospital was 120 minutes [21]. This difference between injury occurrence and notification time can argue for the importance of evaluation of the notification interval in Iranian settings through establishment of injury surveillance [22], which currently is underway.

### Limitation and strength of the study

Accuracy when filling in the data on the questionnaire is one issue. However, data are collected by the trained personnel in the EMS and authors recommend quality control on the data collection process. The future limitation of the study concerns focusing on time intervals and not on quality of care. From the data at hand we could focus on that part of data rather than the severity of the injury and a lack of analysis of potential correlations between injury type and severity. However, they were not within the scope of this study. Another limitation concerns the removal of 187 cases from the list, because of their incomplete data for analysis. We critically tested using the available information at hand and there was no skew and significant variation between this data with that presented in the tables. A strength of this study was that, to our knowledge, it was the first in the country that compared city and interurban roads and the associations and differences for various time intervals, using all cases for a period of two years.

## Conclusion

All time intervals among accident victims on interurban road areas are longer than those for city areas. The short response interval and transport interval compared to other settings may indicate improvement in the pre-hospital services in the study area. It is important for more attention and care to be paid to the notification interval which should be measured by means of the establishment of a surveillance system at the EMS. A public education campaign to increase cooperation is also an important recommendation of this study. Finally, this study implies the need for investigation of the need-for and access-to emergency medical services [15,25].

## Competing interests

The authors declare that they have no competing interests.

## Authors' contributions

MB has made substantial contributions to the conception and design of the study, and has taken responsibility for and coordinated the acquisition of data, which she gathered and analyzed. She took an active part in the analysis of the data, in its abstraction and in the writing-up of the manuscript. DKZ contributed to the conception and design of the study, data collection process and took an active part in the data analysis and results interpretation and writing-up the manuscript. RM also took part in the writing-up and finalization of the manuscript. All authors read and approved the final manuscript.

## Pre-publication history

The pre-publication history for this paper can be accessed here:

http://www.biomedcentral.com/1471-2458/10/406/prepub
